# Covariate-adjusted analysis of the Phase 3 REFLECT study of lenvatinib versus sorafenib in the treatment of unresectable hepatocellular carcinoma

**DOI:** 10.1038/s41416-020-0817-7

**Published:** 2020-04-08

**Authors:** Andrew Briggs, Bruno Daniele, Katherine Dick, Thomas R. Jeffry Evans, Peter R. Galle, Richard A. Hubner, Carlos Lopez, Uwe Siebert, Gabriel Tremblay

**Affiliations:** 1 Department of Health Services Research & Policy, London School of Hygiene and Tropical Medicine, 15-17 Tavistock Place, London, WC1H 9SH UK; 2 Oncology Unit, Ospedale del Mare, Napoli, Italy; 3 Avalon Health Economics LLC, Morristown, NJ USA; 4 University of Glasgow, Glasgow, UK; 5 Medical Department, University Medical Center Mainz, Mainz, Germany; 6 The Christie NHS Foundation Trust, Manchester, UK; 7 Hospital Universitario Marqués de Valdecilla, IDIVAL, Santander, Cantabria Spain; 8 Department of Public Health, Health Services Research and Health Technology Assessment, UMIT – University for Health Sciences, Medical Informatics, and Technology, Hall i.T., Hall in Tirol, Austria; 9 Department of Health Policy and Management, Harvard Chan School of Public Health and Dept of Radiology, Harvard Medical School, Boston, MA USA; 10 Division of HTA, ONCOTYROL—Center for Personalized Cancer Medicine, Innsbruck, Austria; 11 Department of Health Economics and HTA, Purple Squirrel Economics, New York, NY USA

**Keywords:** Outcomes research, Molecularly targeted therapy

## Abstract

**Background:**

In the Phase 3 REFLECT trial in patients with unresectable hepatocellular carcinoma (uHCC), the multitargeted tyrosine kinase inhibitor, lenvatinib, was noninferior to sorafenib in the primary outcome of overall survival. Post-hoc review revealed imbalances in prognostic variables between treatment arms. Here, we re-analyse overall survival data from REFLECT to adjust for the imbalance in covariates.

**Methods:**

Univariable and multivariable adjustments were undertaken for a candidate set of covariate values that a physician panel indicated could be prognostically associated with overall survival in uHCC. The values included baseline variables observed pre- and post-randomisation. Univariable analyses were based on a stratified Cox model. The multivariable analysis used a “forwards stepwise” Cox model.

**Results:**

Univariable analysis identified alpha-fetoprotein (AFP) as the most influential variable. The chosen multivariable Cox model analysis resulted in an estimated adjusted hazard ratio for lenvatinib of 0.814 (95% CI: 0.699–0.948) when only baseline variables were included. Adjusting for post-randomisation treatment variables further increased the estimated superiority of lenvatinib.

**Conclusions:**

Covariate adjustment of REFLECT suggests that the original noninferiority trial likely underestimated the true effect of lenvatinib on overall survival due to an imbalance in baseline prognostic covariates and the greater use of post-treatment therapies in the sorafenib arm.

**Trial registration:**

Trial number: NCT01761266 (Submitted January 2, 2013).

## Background

In a Phase 3 trial (REFLECT) in patients with unresectable hepatocellular carcinoma (uHCC), the multitargeted tyrosine kinase inhibitor, lenvatinib, was shown to be noninferior to the standard-of-care treatment, sorafenib, in terms of the primary outcome of overall survival.^1^ The study reported that the median overall survival was longer for lenvatinib (13.6 months) compared to 12.3 months for sorafenib, but this difference was not statistically significant. The hazard ratio from a stratified Cox proportional hazards model was 0.92 with a 95% confidence interval (CI) of 0.79 to 1.06. Lenvatinib passed the noninferiority test that the upper CI of the hazard ratio for overall survival should be no greater than 1.08.^1^

Although superiority for lenvatinib in terms of overall survival cannot be shown based on the primary efficacy analysis, there are a number of reasons why lenvatinib may be superior to the standard-of-care treatment, sorafenib. First, the superiority of lenvatinib based on secondary end points of progression-free survival, with a reported hazard ratio in the Phase 3 trial of 0.66 (95% CI: 0.57–0.77), and objective response rate, with a reported odds ratio of 3.13 (95% CI: 2.15–4.56). Second, an imbalance in the baseline prognostic factors appeared to bias the outcomes against lenvatinib. Finally, there was a greater number of post-treatment therapies used after sorafenib compared with lenvatinib, leading the authors of the original study to speculate that: “If post-progression survival is prolonged by…post-study treatments, this could lead to a dilution of the observed overall survival treatment benefit”.^1^

The aim of this manuscript is to assess an alternative analysis of the overall survival data from REFLECT to identify and adjust for the imbalance in covariates.

## Methods

REFLECT was a multicentre, randomised, open-label, noninferiority, phase 3 study that compared the efficacy and safety of lenvatinib vs. sorafenib as a first-line systemic treatment in patients with uHCC.^1^ Patients were randomly assigned in a 1:1 ratio to treatment with either lenvatinib 12 mg (if baseline bodyweight was ≥60 kg) or 8 mg (if baseline bodyweight was <60 kg) given once daily orally, or sorafenib 400 mg given twice daily orally. Patients were stratified by: geographical region; presence of macroscopic portal vein invasion (MPVI), extrahepatic spread (EHS) or both; Eastern Cooperative Oncology Group performance status (ECOG PS) 0 or 1; and bodyweight (<60 kg or ≥60 kg).^1^ No crossover to lenvatinib from the sorafenib arm was allowed.

The choice of covariates to consider as candidate variables in this analysis was informed by discussion among the clinical authors of the manuscript, who formed the purposive expert sample.^2^ Each clinician was asked to comment on the likely prognostic importance of the baseline patient characteristics from the original clinical trial,^1^ and all covariates that were considered as potentially important by any of the clinical experts were used in the initial determination of which variables to include in a model for overall survival.

To determine the potential importance of the candidate variables identified by the clinical experts, each variable was entered into the Cox proportional hazards regression model as a univariate adjustment of the treatment effect. This univariate analysis retained the original stratification variables of: geographical region; presence or absence of MPVI, EHS or both; ECOG PS and bodyweight.

A multivariable adjusted analysis was then developed using a forward stepwise procedure from the candidate variables identified by clinicians. In this case, “forwards stepwise” indicates that the procedure starts from a model with treatment effect as the only covariate and systematically considers each candidate variable for inclusion. Candidate covariates were included in further analysis if the *p*-value was <0.05. As variables are added, individual *p*-values for variables in the model can change—in addition to selecting additional variables for inclusion to the model, the stepwise procedure also drops existing variables from the model if the *p*-value becomes >0.1. In the multivariable analysis, the original stratification variables were included as potential covariates, but were not retained as stratification variables.

Sensitivity analyses were conducted on a number of aspects of the analysis: the default Wald test for the standard stepwise procedure was replaced by the Likelihood Ratio (LR) test; variable selection was also tested using the Akaike information criterion (AIC),^3^ which further penalises the likelihood for the number of parameters in the model; and backwards selection (where the model starts with all candidate variables) was contrasted with the forwards selection procedure for determining variable selection. In addition, post-treatment variables were included in the analysis to adjust for the potential dilution effect of the imbalance in post-progression therapies between the two treatment arms (i.e. post-randomisation confounding).

All statistical analyses were undertaken in STATA™ version 14.^4^

## Results

The baseline characteristics for REFLECT, which were considered by the clinical authors to have potential prognostic importance, are presented in Table 1 for the sorafenib and lenvatinib treatment groups. Age and sex are also included for information, although it should be noted that these demographic factors were not considered to impact disease prognosis by the authors. Geographical region (Western vs. Asia-Pacific), MPVI or EHS or both, ECOG PS (0 or 1), and bodyweight (<60 kg or ≥60 kg) were stratification variables in the original clinical trial. Table 1 shows these variables were balanced across the study arms. Some of the non-stratification covariates show potential imbalance: in particular, AFP is known to correlate with prognosis^1,5^ and here the imbalance suggests that the sorafenib arm, with a greater proportion of patients having AFP < 200 ng/ml level, may have included patients with disease who showed better prognosis.^6,7^ There is also evidence that suggests that the effect of sorafenib on overall survival is dependent on patients’ hepatitis status with a greater improvement in survival for sorafenib-treated patients positive for HCV compared to patients positive for HBV, in which sorafenib is less active.^7^ Also included in Table 1 are three post-treatment variables reflecting anti-cancer treatment therapies received post-treatment with either lenvatinib or sorafenib. The post-treatment therapy variable includes both post-treatment anti-cancer procedures (e.g. radiotherapy) and/or post-treatment anti-cancer medications.

**Table 1:** Patient demographics and baseline characteristics.

Characteristic, *n*	Sorafenib (*n* = 476)	Lenvatinib (*n* = 478)
Age, years		
<60	283	270
60–75	126	150
>75	67	58
Sex		
Male	401	405
Female	75	73
Region^a^		
Western	157	157
Asia-Pacific	319	321
Macroscopic portal vein invasion (MPVI)		
Yes	90	109
No	386	369
Extrahepatic spread (EHS)		
Yes	295	291
No	181	187
MPVI, EHS, or both^a^		
Yes	336	329
No	140	149
ECOG Performance status^a^		
0	301	304
1+	175	174
Bodyweight group, kg^a^		
<60	146	153
≥60	330	325
Alpha fetoprotein, ng/ml		
<200	286	255
≥200	187	222
Missing	3	1
Child-Pugh score		
5	357	368
6+	119	110
Number of disease sites		
1	207	207
2	183	167
≥3	86	103
Etiology		
HBV	228	251
HCV	126	91
Alcohol	21	36
Other	32	38
Unknown	69	62
Underlying cirrhosis		
Yes	231	243
No	245	235
BCLC Staging		
Stage B	92	104
Stage C	384	374
Prior procedure		
Yes	344	327
No	132	151
Liver disease site		
Not involved	46	37
Involved	430	441
Lung disease site		
Not involved	332	315
Involved	144	163
Bone disease site		
Not involved	433	427
Involved	43	51
Lymph node disease site		
Not involved	335	351
Involved	141	127
Other disease site		
Not Involved	379	396
Involved	97	82
Post-treatment therapy^b,c^		
Yes	243	206
No	233	272
Post-treatment procedure^b^		
Yes	112	99
No	364	379
Post-treatment medication^b^		
Yes	184	156
No	292	322

*BCLC* Barcelona Clinic Liver Cancer, *ECOG PS* Eastern Cooperative Oncology Group performance status, *HBV* hepatitis B virus, *HCV* hepatitis C virus.

^a^Stratification variables in the original Statistical Analysis Plan.

^b^Post-trandomisation variables.

^c^Post-treatment therapy = post-treatment procedure and/or post-treatment medication.

**Fig. 1:**
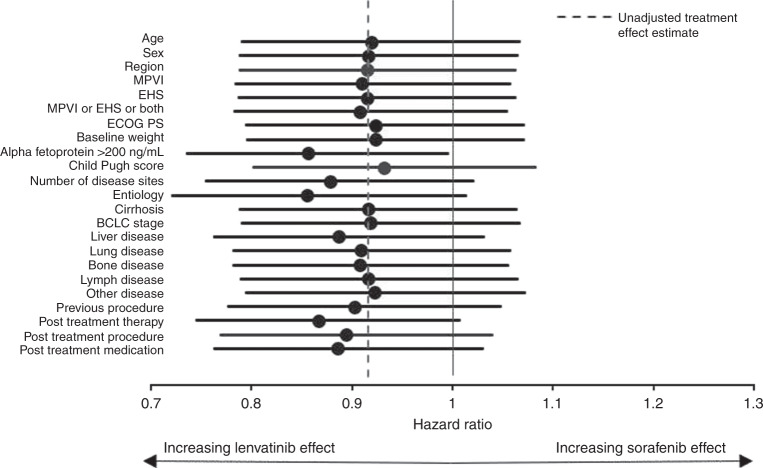
Forest plot of univariate impact of candidate covariates on lenvatinib treatment effect. BCLC Barcelona Clinic Liver Cancer, ECOG PS Eastern Cooperative Oncology Group Performance Status, EHS extrahepatic spread, HBV hepatitis B virus, HCV hepatitis C virus, MPVI macroscopic portal vein invasion.

The Forest plot in Fig. 1 shows the univariable impact on the estimated hazard ratio for lenvatinib treatment compared to sorafenib after adjusting for each covariate in Table 1. In terms of these univariable results, MPVI or EHS or both, AFP < 200 ng/mL, disease site, hepatitis B aetiology, and receipt of a previous procedure are all predictive of overall survival and adjusting for them influences the estimated hazard ratio of the treatment effect in favour of lenvatinib. The Child-Pugh score is also predictive for overall survival, but adjustment favours sorafenib. Finally, adjustments for the imbalance in the post-treatment covariates also have a strong impact on the treatment hazard ratio in favour of lenvatinib. Overall, it is clear from the univariable analysis that AFP level has the single greatest impact on the estimated hazard ratio for overall survival. Indeed, adjusting for AFP alone generates a significant treatment effect in favour of lenvatinib at conventional statistical significance levels with an estimated overall survival hazard of 0.856 with 95% CI ranging from 0.736 to 0.995.

A parsimonious multivariable model is reported in Table 2 using a forward stepwise procedure. Model 1 is the standard model that employs a Wald test for significance. Model 2 represents a sensitivity analysis where the LR test is used instead of the Wald test. As can be seen in Table 2, the final variable selection is identical. In the final column in Table 2, the AIC analysis shows that each successive covariate included in the model further reduces the AIC (i.e. all covariates contribute to the model even when penalising for the use of additional parameters). The confluence of these approaches suggests a strong basis for the multivariable adjusted hazard ratio for lenvatinib overall survival of 0.814 (95% CI: 0.699–0.948).

**Table 2:** Forwards stepwise selection model^a^.

Parameter	Model 1				Model 2				AIC
	HR	*p*-value	95% CI		HR	*p*-value	95% CI		
Lenvatinib	0.814	0.008	0.699	0.948	0.814	0.008	0.699	0.948	8678
Alpha fetoprotein >200 ng/ml	1.725	<0.001	1.478	2.014	1.725	<0.001	1.478	2.014	8603
Child-Pugh Score	1.676	<0.001	1.411	1.992	1.676	<0.001	1.411	1.992	8573
EHS	1.248	0.039	1.011	1.540	1.248	0.039	1.011	1.540	8551
Liver disease involvement	2.345	<0.001	1.676	3.282	2.345	<0.001	1.676	3.282	8518
MPVI	1.351	0.001	1.129	1.617	1.351	0.001	1.129	1.617	8510
Hepatitis B	1.231	0.008	1.057	1.433	1.231	0.008	1.057	1.433	8504
Bone disease involvement	1.510	0.001	1.181	1.932	1.510	0.001	1.181	1.932	8499
Lung disease involvement	1.319	0.005	1.085	1.603	1.319	0.005	1.085	1.603	8496
Other disease involvement	1.289	0.014	1.052	1.579	1.289	0.014	1.052	1.579	8492

*P*  <  0.05 required for covariate inclusion, *P* < 0.1 required for covariate deletion. Model 1 uses the Wald test for inclusion/deletion; Model 2 uses the likelihood ratio test.

*AIC* Akaike information criterion, *EHS* extrahepatic spread, *HR* hazard ratio for overall survival, *MVPI* macroscopic portal vein invasion.

^a^Forwards stepwise selection model starting from model with treatment alone.

A sensitivity analysis is shown in Table 3 for including post-treatment variables into the model. Model 1 includes the composite post-treatment “therapy” variable and Model 2 shows this split out as post-treatment procedures and post-treatment medications. Both models show that the post-treatment anti-cancer variables are highly significant predictors of outcome. In addition, adjusting for these post-randomisation imbalances impacts the hazard ratio of the treatment arms. Comparing the AIC scores for non-nested models shows support for Model 2 compared with Model 1 despite the additional parameter. This gives an adjusted hazard ratio for lenvatinib overall survival of 0.765 (95% CI: 0.656–0.892).

**Table 3:** Sensitivity analysis results adding post-trandomisation covariates.

Parameter	Model 1				Model 2			
	HR	*p*-value	95% CI		HR	*p*-value	95% CI	
Lenvatinib	0.760	<0.001	0.652	0.887	0.765	0.001	0.656	0.892
Alpha fetoprotein >200 ng/ml	1.696	<0.001	1.453	1.980	1.685	<0.001	1.443	1.968
Child-Pugh Score	1.590	<0.001	1.338	1.890	1.579	<0.001	1.328	1.878
EHS	1.248	0.038	1.013	1.538	1.258	0.031	1.021	1.549
Liver disease involvement	2.371	<0.001	1.693	3.319	2.369	<0.001	1.692	3.317
MPVI	1.299	0.004	1.085	1.556	1.309	0.003	1.094	1.567
Hepatitis B	1.222	0.010	1.049	1.422	1.219	0.011	1.047	1.419
Bone disease involvement	1.561	<0.001	1.220	1.997	1.541	0.001	1.205	1.970
Lung disease involvement	1.358	0.002	1.118	1.649	1.358	0.002	1.119	1.649
Other disease involvement	1.229	0.045	1.005	1.504	1.204	0.071	0.984	1.474
Post-treatment therapy	0.599	<0.001	0.514	0.699	–	–	–	–
Post-treatment medication	–	–	–	–	0.681	<0.001	0.579	0.802
Post-treatment procedure	–	–	–	–	0.690	<0.001	0.569	0.835
AIC	8402				8400			

*AIC* Akaike information criterion, *EHS* extrahepatic spread, *HR* hazard ratio for overall survival, *MVPI* macroscopic portal vein invasion.

As an additional sensitivity analysis, the forwards stepwise procedure was repeated as a backwards procedure (Table 4). Here, Model 1 relates to baseline variables only and Model 2 includes post-treatment variables. The backwards selection procedure identifies slightly different adjustment variables than the forward selection process, but the main effects of the model are similar with an adjusted hazard ratio for lenvatinib of 0.814 (95% CI: 0.699–0.948). Similarly, the addition of post-treatment procedure and medication variables gives a virtually identical hazard ratio to the forward selection model: 0.765 (95% CI: 0.656–0.892).

**Table 4:** Sensitivity analysis using backwards selection.

	Model 1^a^				Model 2^b^				Model 3^c^			
	HR	*p*-value	95% CI		HR	*p*-value	95% CI		HR	*p*-value	95% CI	
Lenvatinib	0.814	0.008	0.699	0.948	0.765	0.001	0.656	0.892	0.760	<0.001	0.652	0.887
Alpha Fetoprotein >200 ng/mL	1.725	<0.001	1.478	2.014	1.685	<0.001	1.443	1.968	1.696	<0.001	1.453	1.980
Child-Pugh Score	1.676	<0.001	1.411	1.992	1.579	<0.001	1.328	1.878	1.590	<0.001	1.338	1.890
EHS	1.248	0.039	1.011	1.540	1.258	0.031	1.021	1.549	1.248	0.038	1.013	1.538
Liver Disease Involvement	2.345	<0.001	1.676	3.282	2.369	<0.001	1.692	3.317	2.371	<0.001	1.693	3.319
MPVI	1.351	0.001	1.129	1.617	1.309	0.003	1.094	1.567	1.299	0.004	1.085	1.556
Hepatitis B	1.231	0.008	1.057	1.433	1.219	0.011	1.047	1.419	1.222	0.010	1.049	1.422
Bone Disease Involvement	1.510	0.001	1.181	1.932	1.541	0.001	1.205	1.970	1.561	<0.001	1.220	1.997
Lung Disease Involvement	1.319	0.005	1.085	1.603	1.358	0.002	1.119	1.649	1.358	0.002	1.118	1.649
Other Disease Involvement	1.289	0.014	1.052	1.579	1.204	0.071	0.984	1.474	1.229	0.045	1.005	1.504
Post-treatment Therapy	–	–	–	–	–	–	–	–	0.599	<0.001	0.514	0.699
Post-treatment Medication	–	–	–	–	0.681	<0.001	0.579	0.802	–	–	–	–
Post-treatment Procedure	–	–	–	–	0.690	<0.001	0.569	0.835	–	–	–	–
AIC	8443				8400				8402			

*MVPI* Macroscopic Portal Vein Invasion, *EHS* Extra-Hepatic Spread, *AIC*  Aikake’s Information Criterion.

^a^Backwards selection model starting from full multivariable model. Pr(0.05) required for covariate deletion.

^b^Baseline covariates as Model 1 + post-treatment covariates separately.

^c^Baseline covariates as Model 1 + post-treatment covariates as a composite variable.

## Discussion

This study has reanalysed the REFLECT noninferiority study that compared lenvatinib and sorafenib for the treatment of uHCC in a multivariable modelling framework. The initial study’s strong positive result in favour of lenvatinib for the secondary end points of progression-free survival and objective response rate, the imbalance of baseline covariates favouring sorafenib, and the greater use of subsequent anti-cancer therapies after progression in the sorafenib arm, all suggested that lenvatinib could be superior to sorafenib in terms of overall survival. Our covariate-adjusted results indicate that lenvatinib treatment of uHCC may reach superiority for overall survival vs. sorafenib at conventional levels of statistical significance once baseline imbalances in important prognostic variables are controlled for. This effect is magnified when further adjustment is made for post-treatment therapy variables.

However, we remain cautious when it comes to interpretation of this analysis. This is a post-hoc analysis and cannot change the results of the original trial, though it allows us to better understand them. In particular, adjusting for post-treatment variables violates the randomisation principle and could, itself, lead to bias. More sophisticated techniques and further post-randomisation data on the reasons for receiving the different post-progression therapies would be needed to perform a full causal analysis, adjusting for imbalances in line therapies.^8,9^

By contrast, the use of covariate analysis in randomised controlled studies is far less contentious. Although not permitted in regulatory analyses designed to support licensing applications, the use of covariate adjustment is recommended by leading medical statisticians.^10,11^ This is because covariate adjustment can improve the analysis both in terms of correcting for the potential bias of imbalance in prognostic variables and also in terms of increasing the precision of estimated treatment effects. The latter effect occurs even when there is no imbalance if important prognostic variables are available because the multivariable model uses the covariate information to explain some of the “noise” in the data, increasing the precision of all estimated quantities. Note that it is the strength of the prognostic power of the imbalanced variable, rather than the statistical significance of the imbalance that is the issue. Leading medical journals, including *The Lancet*, where the original clinical trial was published,^1^ discourage the use of *p*-values to compare the balance of the randomised groups. Assuming appropriate randomisation, any observed imbalance occurs by chance and so arbitrary *p*-values are not required to make this judgement. Further, it is the prognostic importance of a given variable that determines the importance of any imbalance. This is illustrated in the analysis presented here by noting that adjusting for the imbalance of AFP level had the greatest single effect on the estimated hazard ratio for lenvatinib treatment. However, statistical testing of the imbalance results in a marginally insignificant difference between the groups in Table 1 (*p*-values not reported).

Indeed, a recent issue of *The American Statistician* is heralding the end of the *p*-value and significance testing.^12^ While this may be premature for appropriately conducted, randomised, controlled trials designed to test a specific hypothesis, the principles do apply to secondary analyses such as presented in this manuscript. For this reason, we focus throughout on the estimated treatment effect with accompanying CIs.

## Conclusions

Covariate adjustment of the REFLECT data strongly suggests that the original noninferiority trial likely underestimated the true effect of lenvatinib on overall survival due to imbalances in baseline prognostic covariates (in particular, AFP level) and the comparatively greater use of post-treatment anti-cancer therapies in the sorafenib arm. While the scale of the impact of covariate adjustment on treatment effect varies, the analyses reported here all favoured lenvatinib. Considering the potential biases associated with adjusting for post-randomisation variables, the preferred base-case hazard ratio is that based on adjusting for baseline covariates only.

## Data Availability

Data are held by the sponsors: Eisai Inc., and Merck Sharp & Dohme Corp., a subsidiary of Merck & Co., Inc., to whom all requests for data access should be addressed.
